# Caregiver Self-Regulation as a Key Factor in the Implementation Potential of Caregiver-Mediated Interventions

**DOI:** 10.3390/bs15101336

**Published:** 2025-09-29

**Authors:** Sarah R. Edmunds, Maya Renaud, Nada M. Goodrum, Jessica Bradshaw, Daniel K. Cooper, Brooke Ingersoll

**Affiliations:** 1 Department of Psychology, McCausland College of Arts and Sciences, University of South Carolina, Columbia, SC 29208, USA; 2 Department of Psychology, College of Social Science, Michigan State University, East Lansing, MI 48824, USA

**Keywords:** self-regulation, community-based interventions, implementation outcomes, inner context, behavior change theories, caregiver-mediated intervention, parenting

## Abstract

Caregiver self-regulation may be a critical component of caregivers’ effective delivery of caregiver-mediated interventions (CMIs). CMIs are a highly evidence-based group of interventions that target a broad range of challenges, including social communication, emotion regulation, and externalizing behaviors, for autistic and neurotypical children. CMIs teach caregivers to be “coaches” to help their children learn and practice skills in daily life. However, being a good “coach” likely requires caregivers to optimally self-regulate their emotions, thoughts, and behaviors when working with their children in moments that are often emotionally heightened. Caregiver self-regulation is a set of skills that promote parenting autonomy and confidence: self-sufficiency, self-efficacy, self-management, personal agency, and problem solving. This conceptual paper will briefly discuss the literature on the role of caregiver self-regulation in CMIs and argue that future implementation research on CMIs should measure caregiver self-regulation because, in line with recent expansion of the theory of planned behavior, caregiver self-regulation may predict more effective implementation of CMIs. We also argue, in line with CFIR 2.0, that supporting caregiver self-regulation could ultimately improve the implementation of CMIs with regard to each implementation outcome in the Implementation Outcomes Framework. For example, enhancing caregiver self-regulation may improve CMI appropriateness (by increasing alignment with each caregiver’s values and culture), adoption (by increasing engagement to finish the full CMI protocol), and even CMI sustainability (by increasing caregivers’ ability to problem-solve and generalize to new child challenges independently, freeing up provider time to work with new caregivers and allowing the agency to provide the CMI for a reduced relative cost). Should future research demonstrate that caregiver self-regulation is an implementation determinant, future implementation strategies may need to include support for caregiver self-regulation, because it may explain or enhance the implementation of CMIs across early intervention and community mental health systems.

## 1. Introduction

Caregiver-mediated interventions (CMIs) are a large group of evidence-based interventions that support children’s social emotional development. The practicality of implementing these time-limited interventions makes them an attractive option for implementation in real-world treatment settings. CMIs help caregivers learn to be “coaches” to help their children learn and practice skills in everyday life. (Note: “parents” and “caregiver” will be used interchangeably throughout.) CMIs target a variety of child outcomes, including social communication (e.g., autism-specialized early interventions such as Project ImPACT or Early Start Denver Model (ESDM)) and emotional or behavioral regulation for both autistic and neurotypical children (e.g., Parent–Child Interaction Therapy (PCIT), Tuning in to Kids, Parent Management Training (PMT), Helping the Noncompliant Child (HNC), RUBI, and AIM HI) (Bearss et al., 2015; Mingebach et al., 2018). Given the multi-level nature of the delivery of these interventions (i.e., provider to caregiver to child), it is important that we investigate not only the effectiveness of CMIs in terms of child outcomes, but also the factors that contribute to caregivers’ successful implementation of CMIs in community service settings, especially in the context of autism.

## 2. Implementation Science and CMIs

By definition, caregivers are pivotal agents in the implementation of caregiver-mediated interventions. This has been noted within the field of implementation science, but not yet fully explored. The Consolidated Framework for Implementation Research (CFIR) 2.0, an implementation science framework that seeks to structure and encourage comprehensive implementation evaluation, acknowledges the potential for an individual to be both the innovation recipient and deliverer. This is the case in CMIs. In their role as the deliverer of a CMI, caregivers play a key role, because they themselves become the agent of implementation in therapeutic and naturalistic settings (Damschroder et al., 2022). In their role as the recipient of a CMI, the CFIR 2.0 characteristics subdomain of “need” explicitly includes an individual’s well-being as a determinant of implementation (e.g., fidelity, use, sustainability). Caregivers of children with autism features experience both greater parenting stress and lower quality of life than caregivers of children with other or no developmental concerns, with evidence that parenting stress predicts, or contributes more strongly to, quality of life for caregivers of children with autism features specifically (Edmunds et al., 2025). Thus, caregivers of autistic children may be most in need of support to both enhance their quality of life and deliver CMIs. Caregivers’ ability to self-regulate may be a key factor to implementation success, as they are the ones who ultimately make the decision about whether the CMI is sustained within their home. Further, caregivers’ successful CMI implementation may influence providers’ perceptions about CMI feasibility and appropriateness for families, which may lead them to support the sustained implementation of CMIs at their agency.

Thus far, prior research on caregiver-related predictors of CMI efficacy or CMI implementation has been limited. Parental stress appears to be the strongest caregiver-related factor predicting (decreased) CMI efficacy (see Shalev et al. (2020) for a systematic review). Parenting self-efficacy and caregiver-child relationship quality have also been found to contribute to CMI efficacy in children with externalizing challenges (e.g., Van Den Hoofdakker et al., 2010). With regard to CMI implementation, pre-existing parenting skills may predict caregivers’ amount of sustained CMI use; in a study of a social communication CMI for toddlers with autism, caregivers’ baseline use of the parenting strategies taught within the intervention (e.g., following the child’s lead) enhanced sustained use of the CMI from post-intervention to follow-up (Shih et al., 2024). While baseline parenting skills and characteristics predict CMI efficacy, caregivers’ skills and capacities may also be determinants of their CMI implementation: within the CFIR 2.0 framework, the “capability” determinant within the characteristics subdomain describes the importance of the innovation deliverer’s (i.e., the caregiver’s) confidence in their ability to make change, self-efficacy, and willingness to commit to the innovation in the face of obstacles (Damschroder et al., 2022). For example, there is empirical evidence for caregiver self-efficacy as a predictor (i.e., determinant) of fidelity: caregivers’ therapeutic self-efficacy, or their perceived competence in delivering an intervention, has been found to predict the quality or fidelity of their CMI implementation with autistic children (Wainer & Ingersoll, 2013).

## 3. Self-Regulation as a Key Factor in the Implementation of CMIs

Given that a range of caregiver factors—parental stress, parenting self-efficacy, parenting skills, and therapeutic self-efficacy—contribute to CMI delivery, this suggests that future research should focus on the role of an overarching caregiver competency, self-regulation, as a factor in both CMI efficacy and implementation. Self-regulation is the ability to manage or modulate one’s own emotions, thoughts, and behaviors (Sanders et al., 2019). Self-regulation has been extensively defined and studied from developmental and neurobiological perspectives (e.g., Karoly, 1993; Strauman, 2017; Barkley, 2001). Because the role of the caregiver in CMIs is to “coach” their child, caregivers may need to optimally self-regulate to successfully use the CMI skills they are taught. Self-regulation may be important at many levels: how effective caregivers are at regulating their attention and mood in general, in the context of parenting, and in the context of using a CMI with their child. Caregivers’ self-regulation can be conceptualized both as an implementation determinant of CMIs (i.e., affecting CMI implementation) and as the goal of an implementation strategy designed to support caregivers in using CMI skills.

As a caregiver using CMIs, there are complex skills that go into decision making when responding to a child’s behavior. Self-regulation is one such core skill. According to prior work, caregiver self-regulation within the context of parenting or caregiving is thought to be composed of five key components: self-efficacy, self-sufficiency, self-management, personal agency, and problem solving (Sanders et al., 2019). Self-efficacy refers to the caregivers’ belief that they can do the CMI and that their efforts can help their child grow and change. Self-sufficiency refers to whether the caregiver has the skills and resources required to have greater independence in parenting later. Self-management involves caregivers setting their own intervention goals and coaching themselves (e.g., self-monitoring) outside of direct instruction. Personal agency involves setting realistic goals that fit with the family’s culture and values, and attributing child improvements to the efforts made by the caregiver. Active problem solving is the ability to address ongoing issues, generalizing the CMI skills they have learned.

### Self-Regulation in CMIs for Autistic Youth

Although some determinants of self-regulation capacity have been identified for caregivers for neurotypical children (Sanders et al., 2019), few studies have measured self-regulation within caregivers with autism, within CMI trials, or examined the role of self-regulation as a determinant of CMI effectiveness and implementation. Caregivers’ self-regulation is influenced by biological and environmental factors including temperament, historical influences, caregiver mental health or substance use problems, and community context (Sanders et al., 2019). Increasing caregivers’ involvement in their child’s interventions may also enhance aspects of self-regulation and decrease stress in both caregivers of autistic (e.g., Iadarola et al., 2017) and neurotypical children (Ansar et al., 2023), which may contribute to the efficacy of CMIs themselves (Mingebach et al., 2018).

Beyond studying the benefits of enhanced self-regulation for individual caregivers using CMIs, researchers have rarely evaluated the effect that enhanced caregiver self-regulation may have on the individual- and system-level implementation of CMIs. We argue that if all caregivers are supported to have better self-regulation while using CMIs, this may ultimately enhance the overall feasibility, acceptability, adoption, and sustainment of CMIs within mental health systems. Often, intervention studies focus on child or intervention outcomes (e.g., social emotional development, reduction in children’s functional impairment) rather than implementation outcomes (e.g., CMI acceptability, adoption, sustainability according to caregivers, providers, and clinical care systems). Implementation outcomes include feasibility, acceptability, appropriateness, fidelity, adoption, implementation cost, penetration, and sustainability (Proctor et al., 2011). We argue that increased caregiver self-regulation may be a predictor of successful CMI implementation. When CMIs are offered within healthcare settings, such as early intervention and community mental health systems, caregivers’ well-being may be pivotal in determining whether all eligible children access and receive high-quality versions of those CMIs.

## 4. Incorporating Self-Regulation into Implementation Theory to Enhance CMI Access in Autism

**Figure 1:**
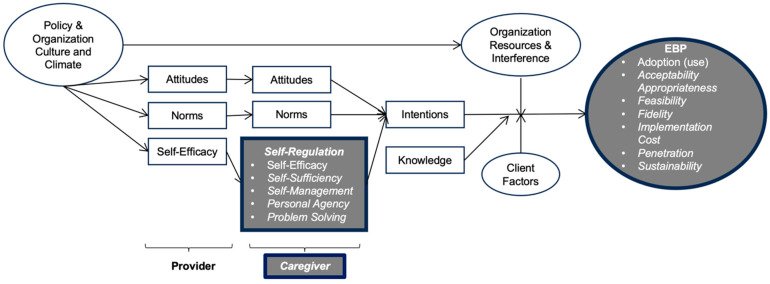
Integration of Caregiver Self-Regulation into Existing Theory as a Key Factor in Supporting Caregiver-Mediated Intervention (CMI) Implementation Outcomes. The theory above is an expansion of the causal theory of EBP use (Becker-Haimes et al., 2021), which is itself an integration of the theory of planned behavior [boxes] and organizational theory [ovals]. We propose to expand the theory by adding the following elements (grey, with thicker borders): expanding the singular original EBP implementation outcome, use/adoption, to include Proctor and colleagues’ entire implementation outcome framework in italics (Proctor et al., 2011); adding a caregiver level of delivery (italics), as CMIs rely on caregivers to learn skills from providers that they then apply with their children; and finally, expanding the original concept of self-efficacy to self-regulation more broadly (italics). We argue that caregivers’ self-regulation is a key client factor that may impact whether or not a CMI will be implemented effectively. EBP = evidence-based practice or intervention.

In line with a recent implementation science-focused expansion of the theory of planned behavior (Becker-Haimes et al., 2021), we propose that caregiver self-regulation may explain or enhance the equitable implementation of CMIs across child-focused service systems. Becker-Haimes and colleagues identify both inner context (e.g., provider, agency, or system in which the CMI is being used) and outer context (e.g., state, national or macro-level system and policy) factors that influence the use of evidence-based interventions as well as individual characteristics of the person delivering them. According to this theory, self-efficacy (i.e., here defined more generally as the belief in one’s ability to implement learned strategies in daily life), along with attitudes and norms, impacts an individual’s intention formation (Becker-Haimes et al., 2021). We propose an expansion of this theory by adding the following elements: (a) expanding the singular original system-level implementation outcome—use/adoption—to include additional implementation outcomes; (b) adding determinants of implementation at the caregiver level of delivery to the existing provider-level determinants, as CMIs rely on caregivers to learn skills from providers that they then apply with their children; and finally, (c) expanding the original concept of general self-efficacy to include all of the theoretical components of caregiver self-regulation (Figure 1). Further, CFIR 2.0 makes the distinction that client factors are innovation determinants, while deliverer factors are implementation determinants (Damschroder et al., 2022). We argue that because caregivers can be conceptualized as CMI deliverers within the CFIR 2.0 framework, their self-regulation is therefore a key implementation determinant that impacts whether or not a CMI will be implemented effectively.

## 5. Caregiver Self-Regulation May Enhance or Explain Implementation Outcomes

Below, we propose the ways in which caregiver self-regulation may improve CMI implementation for a family, which may, ultimately, impact therapist- or system-level implementation for all eight of Proctor and colleagues’ implementation outcomes (2011): acceptability, appropriateness, adoption, fidelity, feasibility, implementation cost, penetration, and sustainability (Table 1). While caregiver self-regulation is likely not an equally powerful determinant of every implementation outcome at each of the individual, provider and healthcare system levels, we offer hypotheses and initial justifications to this effect for the purpose of sparking continued innovation and future research in this area.

If the self-management and self-sufficiency components of self-regulation are targeted within a CMI, the therapist will have guided caregivers to select the goals they address themselves, and caregivers may be more likely to find CMIs *acceptable*. Caregivers with higher or more supported self-sufficiency may also find a CMI more acceptable, because addressing self-sufficiency may bolster the belief that they can address any new child challenges after the intervention ends. Cultural norms, values, and caregivers’ goals for treatment—all of which are emphasized when a CMI is delivered with attention to caregiver self-regulation (Sanders et al., 2019)—may impact the perceived *appropriateness* of a CMI by developing caregivers’ motivation to learn and perform the behavioral requirements of the intervention. An intervention that deviates too far from family values or cultural norms may also influence a caregiver’s likelihood of *adopting* the intervention and their commitment to engaging in its full application. If caregivers are more self-regulated, they may be more likely to try the CMI skills outside of session (e.g., Shih et al., 2024). Caregivers’ confidence and self-efficacy may increase their commitment to full intervention delivery, which would increase the likelihood that the CMI is being implemented with *fidelity* (e.g., Wainer & Ingersoll, 2013). As caregivers continue to make the connection between their actions and the desired changes in their child’s behavior (i.e., personal agency), their perception of the *feasibility* of the CMI—for instance, the caregivers’ feeling that they can problem-solve when using the intervention—may increase as well.

Caregiver self-regulation encourages independent problem solving, thereby improving their ability to generalize CMI skills to other child challenges. If providers then witness caregivers flexibly applying the CMI skills they were taught, and it may increase provider self-efficacy and encourage other providers within the organization to offer the CMI to additional caregivers (*penetration*). If caregivers have learned to generalize strategies and problem solve when faced with challenges in the future, this may reduce the *implementation cost* per family by reducing their need to re-enter active CMI treatment and release providers to work with new families (also enhancing penetration of the CMI within the agency and/or system). Finally, because self-regulation encourages independent problem solving and therefore potentially requires fewer CMI sessions and increases the speed of effective CMI delivery per family, CMIs may ultimately be more *sustainable* for a healthcare system to offer, because they may be able to serve more families per provider.

## 6. Conclusions

While the hypothesis that caregiver self-regulation enhances caregiver well-being and child outcomes has been proposed and explored, future research should assess the impact of caregiver self-regulation on CMI implementation outcomes: feasibility, appropriateness, adoption, and sustainability, among others. Caregivers may be crucial to the implementation and sustained impact of CMIs. As both an interventionist and a client in the CMI framework, caregivers are the linchpin responsible for the success of a CMI. Caregivers of autistic children are especially in need of support, and CMIs are a common format of intervention for autistic children (Edmunds & Hock, 2024). Caregivers’ ability to self-regulate may be essential not only in delivering a CMI to fidelity, but also in ensuring its fit and long-term sustainability. If future research finds that self-regulation is a determinant of certain CMI implementation outcomes, this may necessitate further research on implementation strategies to promote caregivers’ self-regulation (e.g., of their emotions, problem solving, and decision making in the context of parenting and using the CMI skills in particular). For example, when providers use the implementation strategy of high-quality caregiver coaching, caregivers’ therapeutic self-efficacy improved in the context of a social communication CMI for autistic children (Frost & Ingersoll, 2025). Other implementation strategies may enhance other aspects of self-regulation: mindfulness strategies may enhance caregivers’ emotion regulation and personal agency, and outside-of-session reminders and resources may enhance caregivers’ self-management as they use CMI strategies with their child. CMIs have a strong evidence base for improving both autistic and neurotypical child mental health, but their system-level implementation needs improvement. Systems of care (e.g., public mental health or developmental disability agencies, hospital systems) are pressured to select and offer interventions, including CMIs, that are both effective and easily implementable (e.g., Beidas et al., 2016). Supporting caregiver self-regulation during CMIs on an individual level may have cascading effects, ultimately improving the implementation outcomes for CMIs on a broader scale.

## Figures and Tables

**Table 1:** Examples of How Caregiver Self-Regulation May Enhance or Explain Implementation Outcomes.

Implementation Outcome (Proctor et al., 2011)	Hypothesized Effect of Caregiver Self-Regulation on This Outcome Within CMIs
Feasibility *The extent to which the CMI is easy or possible to use*	Self-regulation may enhance caregivers’ belief that they can problem-solve when using the CMI, making it seem more feasible. If caregivers find the CMI feasible, providers may need to engage in less troubleshooting with their clients and might find the overall process of delivering the CMI more feasible as well.
Acceptability *The extent to which the CMI is agreeable or satisfactory*	If the self-management and self-sufficiency components of self-regulation are targeted, caregivers may find CMIs more acceptable, because they are addressing goals that they select themselves and are able to address any other new child need in the future.
Appropriateness *The extent to which the CMI is relevant or compatible*	Empowering self-management and personal agency may enhance the alignment of the CMI with caregivers’ family norms, values, culture, which may lead caregivers, providers, and systems to find the CMI more appropriate.
Fidelity *The extent to which the CMI is delivered as intended*	Caregivers’ increased self-efficacy may enhance their ability to deliver the CMI as intended (e.g., by problem solving their challenges with the CMI outside of session and continuing to use the CMI skills). If more caregivers within the service system use the CMI to fidelity, then system-level fidelity (i.e., average fidelity) may increase.
Adoption *The intention or act of using the CMI*	A focus on caregiver self-regulation may enhance caregivers’ engagement in finishing the full intervention protocol by enhancing fit with caregiver problem solving style or culture, which may lead to more attempts to try CMI skills in daily life, which may lead to better adoption of CMIs across caregivers on average at the system level.
Implementation Cost *The cost (often relative to the status quo) of implementing the CMI*	Increasing caregiver self-regulation during initial CMI delivery may decrease the need for the provider/agency to provide additional intervention sessions, if caregivers have learned to generalize strategies and problem solve on their own.
Penetration *The integration of the CMI within a clinical setting*	Caregivers with better self-regulation may have better independent problem solving (a component of self-regulation), freeing up more agency providers to work with new caregivers, and encouraging more providers to try the CMI within each agency.
Sustainability *The extent to which the CMI is used or maintained during typical practice*	Increased active problem solving may increase the amount of time that caregivers use CMI skills after initial delivery ends, reducing the need for further sessions and freeing providers to work with new families. CMIs may be therefore more sustainable for a healthcare system to offer.

Notes: Italics are used to denote definitions of each implementation outcome as described in Proctor et al. (2011). Self-regulation (as defined by Sanders et al., 2019) is composed of self-efficacy, self-sufficiency, self-management, personal agency, and active problem solving. CMI = caregiver-mediated intervention.

## Data Availability

No new data were created or analyzed in this study. Data sharing is not applicable to this article.

## Abbreviations

The following abbreviations are used in this manuscript:

CMI	Caregiver-Mediated Intervention
CFIR	Consolidated Framework for Implementation Research
EBP	Evidence-Based Practice
ESDM	Early Start Denver Model
PCIT	Parent–Child Interaction Therapy
PMT	Parent Management Training
HNC	Helping the Noncompliant Child
RUBI	Research Units in Behavioral Intervention
AIM HI	An Individualized Mental Health Intervention for Autism
